# Dr. Patterson's Case of Angina Pectoris

**Published:** 1803-07-01

**Authors:** W. Patterson

**Affiliations:** Londonderry


### To Dr. BRADLEY.

DEAR SIR,

THE defective state of our knowledge in several important points relative to the causes and cure of that interesting disease, which goes under the denomination of

*Angina*

*Angina Pectoris*, leads me to offer for the Medical and Physical Journal a brief history of a very characteristic case, in expectation that it may tend to call the attention of your readers to the subject, and thus open a field for disquisition and improvement.

I therefore proceed to communicate the case of H—— E——, Esq. aged sixty-eight years, of a spare make, pale countenance, the prime of his life passed in an active military department, fond of company, but not habitually prone to intemperance, and never sensible of any arthritic affection. During a number of years, he was liable, at uncertain periods, to stomach sickness, attended with a degree of syncope, from which he was occasionally revived by a recumbent posture; yet, in this early stage, some time elapsed before the debilitating effects of the fit were surmounted. Two years, at least, prior to his death, (which happened *suddenly* about three o'clock, p. m. the 21st of May, 1803,) the faintish disposition gradually increased, and changed into a sense of suffocation. After this change took place, the impediment in respiration became, at each successive attack, more strangulating; and at length was excited by very moderate motion on foot; sometimes, if the motion were sudden, even on the most level surface, but not by exercise in a carriage, nor on horse-back.

The evening preceding his death, he had walked out easily, and was arrested by a paroxysm, which continued a considerable time, and left a more morbid condition than usual. Between nine and ten o'clock next morning, I was called to visit him, and found him in bed, complaining chiefly of a pain in the centre of the strenum, accompanied with pains affecting both arms, which he referred to the insertions of the deltoid muscles, or those of the coraco-brachiales. His pulse was unequal, slow, and much disturbed by remarkable pauses.

Previous to my visit he had taken some laxative pills, prescribed by an eminent physician in Dublin, soon after taking which he grew sick, and whilst I sat by him he vomited, but had not any degree of dyspnœa, nor other visible derangement of the respiratory functions. I proposed a blister to the breast, in which he readily acquiesced, as it was an application that, he said, gave immediate relief, several years ago, when he was seized with an acute pain in any part of the thorax; but the blister had not time to operate, before the fatal termination arrived.

Several months antecedent to my visit, he had been under the direction of the physician above alluded to, and had had submitted to the prescriptions of certain inferior practioners, who, as well as I could learn, had advised spirituous aromatic bitters, aloetic laxatives, and *one* issue in the thigh, which was so injudiciously regulated, that it could not be endured longer than two or three weeks. By some of those gentlemen, sulphuric ether was prescribed, to be employed during the paroxysm; but it was used generally with the effect of exciting a discharge from the stomach, and without gaining any curative step.

Three or four months before his death, he had the misfortune to be deprived of a lucrative appointment in the revenue; a misfortune which, doubtless, considering the nature of his malady, conspired to hasten his end. As to the precipitancy of the event, his friends were warned of it; for, on being casually told of his complaints, before I was professionally consulted, I candidly told them, that I was confident his life was not worth an hour's purchase. Some time prior to the catastrophe, he expressed a wish that his body should be opened; but his widow could not bring herself to consent to the operation, and consequently this instructive opportunity was lost.

In order to pursue and promote the object of this communication, it is my intention, as soon as circumstances shall allow, to transmit to you observations touching this case, and on as many publications treating of the same subject, as have come within the reading of,

Yours, &c.

*Londonderry, June* 11, 1803. W. PATTERSON.

